# An Analytical Model for Thermoelastic Damping and Frequency Shift of Micro/Nano Cylindrical Shell Resonators Considering Size-Dependent Effects

**DOI:** 10.3390/mi17060660

**Published:** 2026-05-26

**Authors:** Guoshuai Wang, Pan Liu, Qiang Zhang, Ling Jiang, Chunyan Xia, Jiawei Wang, Houchuan Lai

**Affiliations:** Southwest Institute of Technical Physics, Chengdu 610041, China; pwxuan0520@163.com (G.W.); lp_electronic@163.com (P.L.); jianglingmssj@163.com (L.J.); xcy270104029@163.com (C.X.); wangjv030@163.com (J.W.); lhch43@163.com (H.L.)

**Keywords:** thermoelastic damping, nonlocal dual-phase-lag heat conduction model, nonlocal elasticity theory, micro/nano cylindrical shell, frequency shift

## Abstract

Thermally induced frequency shift (FS) and energy dissipation are key factors limiting the quality factor (Q-factor) of resonators. This study combines nonlocal elasticity theory (NET) with the nonlocal dual-phase-lag (NDPL) heat-conduction model to establish a theoretical framework for evaluating thermoelastic damping (TED) in micro/nano cylindrical shells with size-dependent effects. The equation of motion of the cylindrical shell is simplified using the Donnell–Mushtari–Vlasov (DMV) approximation. The resonant frequency of the cylindrical shell with size-dependent effects is obtained by combining the compatibility equation with the equation of motion and applying the Galerkin method. Additionally, an analytical solution for the TED of cylindrical shells considering size effect under classical boundary conditions is derived using the complex frequency method. The proposed formulation is validated by comparing its predictions with available numerical results. Numerical results indicate that size effects have a significant impact on the TED of cylindrical shells, particularly as mechanical nonlocal effects increase TED, thereby reducing the Q factor of micro/nano cylindrical shells. Moreover, the impact of size effects on the FS and frequency attenuation (FA) is examined. This study lays crucial theoretical groundwork for the design of resonators utilizing micro/nano cylindrical shell materials.

## 1. Introduction

Micro/nano resonators are one of the significant applications in micro/nano electromechanical systems (MEMS/NEMS), with core structures typically composed of advanced materials such as semiconductor [1], metals [2], functionally graded materials [3], carbon nanotubes [4], or graphene [5]. Micro/nano resonators have become key functional components in many modern sensing and actuation systems because their reduced dimensions enable high sensitivity, fast response, and low energy consumption [6]. TED originates from the coupling between elastic deformation and heat conduction: periodic mechanical strain creates temperature gradients, and the resulting irreversible heat flux dissipates vibrational energy. For high-performance resonant devices, thermoelastic damping is undesirable because it introduces intrinsic energy loss and thereby compromises frequency stability and sensing accuracy [7]. External losses, including gas damping and anchor loss, can often be mitigated through vacuum packaging or structural optimization, whereas TED is governed by material deformation temperature coupling inside the resonator [8]. This characteristic cannot be reduced or eliminated merely by changing the boundary conditions of the supporting structure or employing optimization strategies such as vacuum packaging. Therefore, accurately predicting the TED of resonators can provide a scientific basis for their design and application, thereby facilitating further development of micro/nano resonators in high-precision measurement and signal processing fields.

In the early 20th century, Zener [9,10], based on an understanding of the internal friction phenomena in gases, first introduced the concept of TED, positing that intermolecular friction is the fundamental cause of TED, with the corresponding theoretical model known as the Zener model. Additionally, Zener proposed two fundamental methods for predicting TED in structures: the complex frequency method and the thermal energy method [9,10]. Subsequently, Lifshitz and Rouke [11] successfully established a theoretical model for predicting TED in rectangular cross-section beams based on the Euler–Bernoulli beam theory. These two models laid a significant theoretical foundation for academic research in the field of TED.

Research on TED in micro/nano resonator structures has been extensively carried out, encompassing various structural forms and material systems. Common resonator architectures at small scales include slender beams [12,13], planar plates [14,15], annular rings [16,17], and curved shell structures [18,19]. Among these, micro/nano beams, as the most representative form of resonators, have been investigated in a more systematic and in-depth manner regarding their TED behavior. Existing research primarily explores the impact of factors such as beam height [20], cross-sectional geometry [21], boundary conditions [22], and material properties [23] on TED. The analysis of TED in micro/nano plate resonators has also made certain research progress. Due to their unique geometric configuration, annular resonators demonstrate broad application prospects in the fields of sensors and actuators. Related studies focus on the TED behavior of laminated micro rings and circular cross-section micro rings under out-of-plane vibration modes. Such structures are relevant to carbon nanotube resonators, hollow nanowire resonators, cylindrical nano-shell sensors, micro-tube resonators, mass sensors, and high-frequency nanoelectromechanical resonators. In these devices, thermoelastic damping is one of the intrinsic energy dissipation mechanisms that may limit the quality factor. The influencing factors of TED in shell structures, as important components of resonators, have also attracted widespread attention. Lu et al. [24] were the first to establish a theoretical model for TED in cylindrical shell structures, providing an important theoretical foundation for subsequent research. On this basis, Kim and Kim [25] investigated the effects of geometric size, initial stress, and modal order on the correlation between natural frequencies and TED in cylindrical shells. Additionally, the study by Hoseinzadeh and Khade [26] focused on double-walled carbon nanotubes, analyzing how interlayer van der Waals interactions and initial axial stress influence their thermal–elastic vibration and damping characteristics. In summary, the TED behavior of resonator components is influenced by multiple factors, including geometry and size, vibration frequency, material composition, operating temperature, pre-stress state, internal defects, and boundary conditions. It is evident that the mechanisms impacting TED are extremely complex and require further in-depth study to provide theoretical support for optimizing the overall performance of resonators.

Extensive theoretical and experimental studies have shown that Fourier’s law has limitations under certain conditions. It is generally applicable to conventional heat-conduction problems at large spatial scales and long time scales [27] but may fail to accurately describe heat transport under ultrafast or nanoscale thermal processes. These conditions involve short durations, high heat fluxes, and large temperature gradients, such as those encountered in ultrafast laser pulse heating, ultra-low temperatures, and ultra-high temperatures [28]. Concurrently, as the characteristic structural dimensions decrease to the micro/nanoscale, the mechanical properties of materials also undergo significant changes [29]. These changes manifest specifically as material softening or hardening phenomena, as well as alterations in electromechanical coupling characteristics [30]. This phenomenon, which significantly influences material properties at the microscopic scale but can be neglected at the macroscopic scale, is referred to as the “size effect.” It is noteworthy that the classical Fourier heat conduction model and traditional thermoelasticity theory struggle to accurately describe the thermoelastic behavior of materials at the micro/nanoscale. Consequently, classical TED models, which are built upon these theories, also exhibit significant limitations when predicting the thermoelastic damping of micro/nano resonators.

In recent years, scholars have actively explored the influence of size effects on the TED of micro/nano structures. In this field, researchers have adjusted classical TED models by employing NET [31], modified couple stress theory (MCST) [32], strain gradient theory (SGT) [33], and surface elasticity theory (SET) [34] to include size effects at the mechanical level. On the other hand, to better describe the effects of non-Fourier heat conduction on the thermal performance of micro/nano-scale structures, researchers have developed TED models considering wave effects based on the Cattaneo–Vernotte (CV) wave model [35], dual-phase-lags heat conduction (DPL) model [36], NDPL model [37], three-phase-lag (TPL) heat conduction model [38], and Moore–Gibson–Thompson (MGT) heat conduction model [39]. Currently, numerous studies have investigated the specific effects of size effects on the TED of various micro/nano structures, providing an important theoretical foundation for understanding and improving the design of micro/nano resonators. Borjalilo et al. [40,41] combined strain gradient theory with nonlocal elastic theory. They used the DPL heat conduction model to analyze the size dependence of TED in micro/nano beams. Kumar and Mukhopadhyay [42] combined MCST with NET. They explored the size-dependent behavior of TED in micro/nano beams based on the MTG heat conduction model. Anjali and Santwana [43] studied the TED of micro/nano piezoelectric beams based on modified couple stress theory and the DPL heat conduction model. Pen et al. [44] conducted a TED analysis using SET and the NDPL heat conduction model. Their findings show that surface effects enhance the bending stiffness of sandwich micro/nano beams, leading to a decrease in energy dissipation due to thermoelastic coupling. Bashar et al. [45] developed a TED model for micro/nano rectangular plates under three-dimensional heat conduction conditions based on MCST and the DPL heat conduction model. Li et al. [46] established a TED model for micro/nano rectangular plates under three-dimensional heat conduction conditions based on modified couple stress theory and the NDPL heat conduction model. Li et al. [47,48] combined NET with the DPL model and the GK heat conduction model to investigate the effects of key physical quantities, such as mechanical nonlocal parameters and thermal nonlocal parameters, on the TED of micro/nano cylindrical shells. Shi and Fan [49] developed a TED model for micro/nano cylindrical shells based on SET and a DPL heat conduction model. They investigated the impact of thermal wave parameters on TED. In summary, a comprehensive literature review reveals several key issues. First, size effects at the micro/nanoscale have a significant impact on the TED of cylindrical shell structures. Furthermore, the predictions of TED for the same structure vary significantly across different theoretical frameworks. This indicates that the influence of small-size effects on TED is quite complex, and existing theories require further refinement. Unfortunately, due to the complexity of the mechanical governing equations for cylindrical shells, researchers have primarily focused their studies on simplified beams, plates, and rings, with relatively few studies on cylindrical shell structures. Therefore, research on the effects of size at the micro/nanoscale on the TED of cylindrical shell structures is still insufficient, and related issues require further exploration by scholars.

This paper establishes, for the first time, a TED model for cylindrical shells under classical boundary conditions, based on NET and the NDPL model. It aims to investigate the size effect of the TED of micro/nano cylindrical shell resonators. Different from existing cylindrical shell TED models, mainly based on surface elasticity or conventional DPL heat conduction, the present study simultaneously incorporates mechanical nonlocality and nonlocal DPL heat conduction. This treatment enables the separate evaluation of long-range elastic interactions and spatially nonlocal thermal transport on TED and FS. Additionally, it analyzes the main factors affecting the TED of cylindrical shell structures. This provides a theoretical foundation for their engineering applications. In the theoretical modeling process, the simplified vibration equation of cylindrical shells considering nonlocal effects and the NDPL model was derived using the DMV theory. Subsequently, the resonance frequency of the cylindrical shell was determined using the Galerkin method. On this basis, the analytical expression for the TED of the cylindrical shell was derived using the complex frequency method. In the numerical simulation section, the effects of mechanical nonlocal parameters and thermal nonlocal parameters on TED, FS, and FA of the cylindrical shell were discussed in detail. The numerical results indicate that size effects significantly influence the TED of the cylindrical shell.

The content of this paper is organized as follows: The second section first establishes the thermal–elastic vibration equation for micro/nano cylindrical shells based on NET and the NDPL model. Next, the resonance frequency of the cylindrical shell is obtained using the Galerkin method. Finally, the analytical solution for the TED of the cylindrical shell is derived using the complex frequency method. The third section verifies the correctness of the theoretical derivation and numerical simulation of this model through a numerical comparison method. It also investigates in detail the main factors affecting the TED, FS, and FA of cylindrical shells at the nanoscale. The fourth section summarizes the main conclusions of this study.

## 2. Size Effects in Generalized Thermoelasticity Models of Cylindrical Shells

This section uses the DMV approximation to develop a TED model for micro/nano cylindrical shells, integrating NET with non-Fourier heat conduction behavior. This model accounts for mechanical size effects and non-Fourier heat conduction effects to more accurately predict the TED of cylindrical shells at the micro/nanoscale.

### 2.1. Equations of Motion

**Figure 1:**
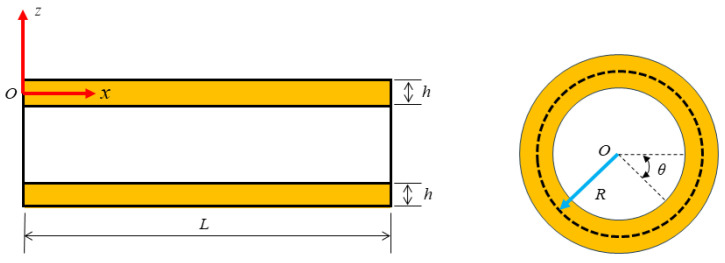
Schematic of a nano cylindrical shell.

The configuration and coordinate system of a thin cylindrical shell with length *L*, radius *R*, and thickness *h* are depicted in Figure 1. According to thin shell theory, the other displacement components satisfy the following relations [24,25,47,48]:

(1a) $$U_{x}=u_{\left(x,\theta ,t\right)}-z\frac{\partial w_{\left(x,\theta ,t\right)}}{\partial x}\;,\;U_{\theta }=v_{\left(x,\theta ,t\right)}-\frac{z}{R}\frac{\partial w_{\left(x,\theta ,t\right)}}{\partial x},$$

(1b) $$U_{z}=w_{\left(x,\theta ,t\right)},$$

where $U_{x}$ represents the displacement along the length direction, $U_{\theta }$ represents the displacement along the circumferential direction, and $U_{z}$ represents the radial displacement. Parameters $u_{\left(x,\theta ,t\right)}$ and $v_{\left(x,\theta ,t\right)}$ stand for the displacement components of the mid-surface in the *x* and θ directions, respectively. For simplicity, the following calculations will set, i.e., $u=u_{\left(x,\theta ,t\right)}$, $v=v_{\left(x,\theta ,t\right)}$, and $w=w_{\left(x,\theta ,t\right)}$.

Based on the above relations, the strain components are as follows [24,25,47,48]:

(2a) $$\varepsilon_{xx}=\varepsilon_{xx}^{0}+zK_{xx},$$

(2b) $$\varepsilon_{\theta \theta }=\varepsilon_{\theta \theta }^{0}+zK_{\theta \theta },$$

(2c) $$\varepsilon_{x\theta }=\varepsilon_{x\theta }^{0}+zK_{x\theta }\;.$$

where $\varepsilon_{xx}$, $\varepsilon_{\theta \theta }$, and $\varepsilon_{x\theta }$ respectively represent the strain in the *x* direction, circumferential strain, and shear strain. Moreover, $\varepsilon_{xx}^{0}=\frac{\partial u}{\partial x}$, $\varepsilon_{\theta \theta }^{0}=\frac{1}{R}\frac{\partial v}{\partial \theta }+\frac{w}{R}$, $\varepsilon_{x\theta }^{0}=\frac{1}{R}\frac{\partial u}{\partial \theta }+\frac{\partial v}{\partial x}$, $K_{xx}=-\frac{\partial^{2}w}{\partial x^{2}}$, $K_{\theta \theta }=-\frac{1}{R^{2}}\frac{\partial^{2}w}{\partial \theta^{2}}$, and $K_{x\theta }=-\frac{2}{R}\frac{\partial^{2}w}{\partial x\partial \theta }$.

Due to the dimension in the thickness direction of the cylindrical shell being much smaller than that in other directions, the normal stress across the thickness direction can be approximately neglected, i.e., *σ_zz_* = 0 [24,25,47,48]. Therefore, the strain along the thickness of the cylindrical shell satisfies the following relationship:

(3) $$\varepsilon_{zz}=\frac{\nu }{\nu -1}\left(\varepsilon_{xx}+\varepsilon_{\theta \theta }\right)+\frac{1+\nu }{1-\nu }\alpha_{T}\Delta T.$$

According to Eringen’s (1983) theory of nonlocal elasticity, the constitutive equation of a cylindrical shell considering thermoelastic coupling is as follows [47,48]:

(4a) $$\left(1-\mu_{0}\nabla^{2}\right)\sigma_{xx}=\frac{E}{1-\nu^{2}}\left(\varepsilon_{xx}+\nu \varepsilon_{\theta \theta }\right)-\frac{E\alpha_{T}}{1-\nu }\Delta T,$$

(4b) $$\left(1-\mu_{0}\nabla^{2}\right)\sigma_{\theta \theta }=\frac{E}{1-\nu^{2}}\left(\nu \varepsilon_{xx}+\varepsilon_{\theta \theta }\right)-\frac{E\alpha_{T}}{1-\nu }\Delta T,$$

(4c) $$\left(1-\mu_{0}\nabla^{2}\right)=\frac{E}{2\left(1+\nu \right)}\varepsilon_{x\theta },$$

where E, ν, and $\alpha_{T}$ represent elasticity modulus, Poisson ratio, and thermal expansion coefficient, respectively. Moreover, the symbol $\nabla^{2}$ stands for the Laplace operator, $\sigma_{xx}$ denotes the stress in the x direction, $\sigma_{\theta \theta }$ represents the circumferential stress, $\sigma_{x\theta }$ is the shear stress, and $\mu_{0}$ represents a nonlocal parameter in mechanics and has the following relationship:

(5) $$\mu_{0}={\left(\tau l\right)}^{2}\;\;with\;\;\tau =\frac{e_{0}a}{l},$$

in which $e_{0}$ is a material constant and *a* and *l* denote the internal and external characteristic lengths, respectively.

Membrane forces are defined via the following relations:

(6) $$N_{ij}=\int_{-\frac{h}{2}}^{\frac{h}{2}}\sigma_{ij}dz,\;i,j=x,\;\theta ,\;\mathrm{and}\;z,$$

Bending moments are defined via the following relations:

(7) $$M_{ij}=\int_{-\frac{h}{2}}^{\frac{h}{2}}\sigma_{ij}zdz,\;i,j=x,\;\theta ,\;\mathrm{and}\;z,$$

Substituting Equations (4a–c) into Equation (6), one can obtain:

(8a) $$\left(1-\mu_{0}\nabla^{2}\right)N_{xx}=\frac{Eh}{1-\nu^{2}}\left(\varepsilon_{xx}^{0}+\nu \varepsilon_{\theta \theta }^{0}\right)-\frac{N_{T}}{1-\nu },$$

(8b) $$\left(1-\mu_{0}\nabla^{2}\right)N_{\theta \theta }=\frac{Eh}{1-\nu^{2}}\left(\nu \varepsilon_{xx}^{0}+\varepsilon_{\theta \theta }^{0}\right)-\frac{N_{T}}{1-\nu },$$

(8c) $$\left(1-\mu_{0}\nabla^{2}\right)N_{x\theta }=\frac{Eh}{2\left(1+\nu \right)}\varepsilon_{x\theta },$$

The thermal membrane force generated by the temperature variation is represented as $N_{T}$. The calculation method is as follows:

(9) $$N_{T}=E\alpha_{T}\int_{-\frac{h}{2}}^{\frac{h}{2}}\Delta Tdz.$$

Substituting Equations (4a–c) into Equation (7), the bending moment can be obtained:

(10a) $$\left(1-\mu_{0}\nabla^{2}\right)M_{xx}=A_{0}\left(K_{xx}+\nu K_{\theta \theta }\right)-\frac{1}{1-\nu }M_{T},$$

(10b) $$\left(1-\mu_{0}\nabla^{2}\right)M_{\theta \theta }=A_{0}\left(\nu K_{xx}+K_{\theta \theta }\right)-\frac{1}{1-\nu }M_{T},$$

(10c) $$\left(1-\mu_{0}\nabla^{2}\right)M_{x\theta }=\frac{A_{0}}{2}\left(1-\nu \right)K_{x\theta },$$

where $A_{0}=\frac{Eh^{3}}{12\left(1-\nu^{2}\right)}$, and $M_{T}$ is the thermal bending moment generated by the temperature variation. The thermal bending moment can be calculated as follows:

(11) $$M_{T}=E\alpha_{T}\int_{-\frac{h}{2}}^{\frac{h}{2}}z\Delta Tdz.$$

According to the DMV approximation, the motion equation of a cylindrical shell in the z direction satisfies the following relationship [24,25,47,48]:

(12) $$\frac{\partial^{2}M_{xx}}{\partial x^{2}}+\frac{2}{R}\frac{\partial^{2}M_{x\theta }}{\partial x\partial \theta }+\frac{1}{R^{2}}\frac{\partial^{2}M_{\theta \theta }}{\partial \theta^{2}}-\frac{N_{\theta \theta }}{R}=\rho h\frac{\partial^{2}U_{z}}{\partial t^{2}},$$

The compatibility equation of cylindrical shells satisfies the following relationship [24,25,47,48]:

(13) $$\frac{\partial^{2}\varepsilon_{\theta \theta }^{0}}{\partial x^{2}}-\frac{1}{R}\frac{\partial^{2}\varepsilon_{x\theta }^{0}}{\partial x\partial \theta }+\frac{1}{R^{2}}\frac{\partial^{2}\varepsilon_{xx}^{0}}{\partial \theta^{2}}+\frac{K_{xx}}{R}=0.$$

This study focuses on thin-walled cylindrical shells primarily subjected to lateral vibrations. For thin shells with small thickness-to-radius ratios and thickness-to-length ratios, lateral bending vibration usually dominates the dynamic response, while the contribution of in-plane inertia is relatively small. Therefore, within the framework of the DMV shell theory, neglecting in-plane inertia can be considered a reasonable first-order approximation. According to the DMV approximation, it is assumed that transverse vibrations dominate and that the effects of in-plane inertial forces are negligible [24,25,47,48]. It should be noted that the lateral displacement corresponds to the normal direction of the shell, not the axial direction. Therefore, the axial and circumferential motion equations of a cylindrical shell satisfy the following relationship [24,25,47,48]:

(14a) $$\frac{\partial N_{xx}}{\partial x}+\frac{1}{R}\frac{\partial N_{\theta x}}{\partial \theta }=0,$$

(14b) $$\frac{\partial N_{x\theta }}{\partial x}+\frac{1}{R}\frac{\partial N_{\theta \theta }}{\partial \theta }=0,$$

In order to find a solution that satisfies Equation (14a,b), the in-plane stress function $\Gamma =\Phi \left(x,\theta \right)e^{j\omega \;t}$ is introduced. The membrane force and plane stress function of a cylindrical shell satisfy the following relationship [24,25,47,48]:

(15) $$N_{xx}=\frac{1}{R^{2}}\frac{\partial^{2}\Gamma }{\partial \theta^{2}},N_{\theta \theta }=\frac{\partial^{2}\Gamma }{\partial x^{2}},N_{\theta x}=-\frac{1}{R}\frac{\partial^{2}\Gamma }{\partial x\partial \theta }.$$

According to Equations (8a–c), one can obtain:

(16a) $$\varepsilon_{xx}^{0}=\frac{1}{Eh}\left(\left(1-\mu_{0}\nabla^{2}\right)N_{xx}-\nu N_{\theta \theta }+N_{T}\right),$$

(16b) $$\varepsilon_{\theta \theta }^{0}=\frac{1}{Eh}\left(\left(1-\mu_{0}\nabla^{2}\right)N_{\theta \theta }-\nu N_{xx}+N_{T}\right),$$

(16c) $$\varepsilon_{x\theta }=\frac{2\left(1+\nu \right)}{Eh}\left(1-\mu_{0}\nabla^{2}\right)N_{x\theta }.$$

Based on Equations (2a,b) and Equation (3), the volumetric strain e can be obtained as follows:

(17) $$e=\frac{1-2\nu }{1-\nu }\left(\left(\varepsilon_{xx}^{0}+\varepsilon_{\theta \theta }^{0}\right)+z\left(K_{xx}+K_{\theta \theta }\right)\right)+\frac{1+\nu }{1-\nu }\alpha_{T}\Delta T.$$

Based on Equations (15) and (16a–c), the volumetric strain e can be re-expressed as:

(18) $$\begin{array}{l} e=\frac{1}{Eh}\frac{1-2\nu }{1-\nu }\left(\left(1-\nu \right)\left(1-\mu_{0}\nabla^{2}\right)\left(N_{\theta \theta }+N_{xx}\right)+2N_{T}\right)\\ \;\;\;-\frac{1-2\nu }{1-\nu }z\;\nabla^{2}U_{z}+\frac{1+\nu }{1-\nu }\alpha_{T}\Delta T, \end{array}$$

where $\nabla^{2}=\frac{\partial^{2}}{\partial x^{2}}+\frac{1}{R^{2}}\frac{\partial^{2}}{\partial \theta^{2}}$ is the Laplacian operator in polar coordinates.

Considering the thermoelastic coupling effect is relatively weak, the magnitude of $N_{T}$ is much smaller than the magnitudes of $N_{xx}$ and $N_{\theta \theta }$. Therefore, the $N_{T}$ in Equation (18) can be neglected [24,25,47,48]. Consequently, the volumetric strain of the cylindrical shell can be further expressed as:

(19) $$e=\left(1-\mu_{0}\nabla^{2}\right)\frac{\left(1-2\nu \right)}{Eh}\nabla^{2}\Gamma -\frac{1-2\nu }{1-\nu }z\;\nabla^{2}U_{z}+\frac{1+\nu }{1-\nu }\alpha_{T}\Delta T.$$

### 2.2. Temperature Field Control Equation

Tzou and Guo [50] established a heat conduction equation considering nonlocal thermal effects, which takes the following form:

(20) $$\left(1+\tau_{Q}\frac{\partial }{\partial t}-l_{Q}^{2}\nabla^{2}\right)q=-\kappa \left(1+\tau_{T}\frac{\partial }{\partial t}\right)\nabla \Delta T.$$

where q represents the heat flow vector, κ denotes the thermal conductivity, $l_{Q}$ indicates the thermal nonlocal parameter, and ∇ represents the Hamiltonian operator. Additionally, $\tau_{Q}$ and $\tau_{T}$ represent the lag times for heat flow and temperature gradient, respectively.

For isotropic materials, the energy conservation equation is expressed as follows [44]:

(21) $$-\nabla q=\rho C_{E}\frac{\partial \Delta T}{\partial t}+\frac{E\alpha_{T}T_{0}}{1-2\upsilon }\frac{\partial e}{\partial t},$$

where $C_{E}$ and ρ represent the specific heat per unit mass and mass density, respectively.

By utilizing Equations (20) and (21), one can derive the thermally coupled equation based on the NDPL model [44]:

(22) $$\kappa \left(1+\tau_{T}\frac{\partial }{\partial t}\right)\nabla^{2}\Delta T=\rho C_{E}\left(1+\tau_{Q}\frac{\partial }{\partial t}-l_{Q}^{2}\nabla^{2}\right)\left(\frac{\partial \Delta T}{\partial t}+\frac{E\alpha_{T}T_{0}}{1-2\upsilon }\frac{\partial e}{\partial t}\right).$$

where ${\hat{\nabla }}^{2}=\frac{1}{R+z}\frac{\partial }{\partial z}+\frac{\partial^{2}}{\partial z^{2}}+\frac{1}{{\left(R+z\right)}^{2}}\frac{\partial^{2}}{\partial \theta^{2}}+\frac{\partial^{2}}{\partial x^{2}}$ is the Laplacian operator in cylindrical coordinates. It is important to emphasize that when non-Fourier heat conduction behaviors are neglected, i.e., when $\tau_{T}=0$, $\tau_{Q}=0$, and $l_{Q}=0$, Equation (22) can degenerate into the temperature field control equation based on the classical heat conduction model.

It should be noted that in actual heat transfer processes, the temperature gradient in the thickness direction is significantly greater than that in other directions. Therefore, the temperature gradient in other directions can be neglected, i.e., ${\hat{\nabla }}^{2}=\frac{1}{R+z}\frac{\partial }{\partial z}+\frac{\partial^{2}}{\partial z^{2}}$ [24,25,47,48,49]. Moreover, due to R≫z for the cylindrical shell, R+z can be replaced with R in Equation (22) [24,25,47,48,49].

The temperature variation ΔT under harmonic vibrations of a cylindrical shell can be written as [24,25]:

(23) $$\Delta T=\Theta_{\left(x,\theta ,z\right)}e^{j\omega t},$$

where $\Delta T=T-T_{0}$, the parameters T and $T_{0}$ are the current temperature and reference temperature, respectively. For simplicity, the following calculations will be set, i.e., $\Theta =\Theta_{\left(x,\theta ,z\right)}$.

Substituting Equations (23) and (19) into Equation (22), one can obtain:

(24) $$\frac{\partial^{2}\Theta }{\partial z^{2}}+\frac{1}{R}\frac{\partial \Theta }{\partial z}+p^{2}\left(1+q\Delta_{E}\right)\Theta =p^{2}\left(\frac{\Delta_{E}\left(1-\mu_{0}\nabla^{2}\right)\nabla^{2}\Phi }{\alpha_{T}Eh}-\frac{\Delta_{E}}{\alpha_{T}\left(1-\nu \right)}z\;\nabla^{2}w\right),$$

where $\Delta_{E}=\frac{T_{0}E\alpha_{T}^{2}}{\rho C_{E}}$, $p=\frac{\xi }{h}\sqrt{\varphi_{1}-\varphi_{2}j}$, $\varphi_{1}=\frac{\omega \left(\tau_{q}-\tau_{T}^{*}\right)}{\left(1+\omega^{2}\tau_{T}^{{*}^{2}}\right)}$, $\varphi_{2}=\frac{\left(\tau_{q}\tau_{T}^{*}\omega^{2}+1\right)}{\left(1+\omega^{2}\tau_{T}^{{*}^{2}}\right)}$, $\xi =h\sqrt{\frac{\omega }{\chi }}$, $\chi =\frac{\kappa }{\rho C_{E}}$, $q=\frac{1+\nu }{\left(1-2\nu \right)\left(1-\nu \right)}$ and $\tau_{T}^{*}=\left(\tau_{T}+\frac{l_{Q}^{2}}{\chi }\right)$.

In fact, owing to the fact that the magnitude of relaxation strength is insignificant in most cases ($\Delta_{E}\ll 1$) [24,25,47,48,49]. It should be noted that in the thermal–elastic damping analysis of high-Q resonators, $\Delta_{E}\ll 1$ is due to the fact that the dissipated thermal–elastic energy within one vibration cycle is much smaller than the stored elastic energy. Nevertheless, for extremely high-frequency nanoscale resonators or cases with strong thermo-mechanical coupling, this assumption may require further verification. Therefore, the heat conduction Equation (23) can be simplified as:

(25) $$\frac{\partial^{2}\Theta }{\partial z^{2}}+\frac{1}{R}\frac{\partial \Theta }{\partial z}+p^{2}\Theta =\Delta_{E}p^{2}\left(\frac{\left(1-\mu_{0}\nabla^{2}\right)}{\alpha_{T}Eh}\nabla^{2}\Phi -\frac{1}{\alpha_{T}\left(1-\nu \right)}z\;\nabla^{2}w\right).$$

The general solution of Equation (24) satisfies the following relationship:

(26) $$\Theta =b_{1}e^{c_{\;1}z}+b_{2}e^{c_{\;2}z}+\left(z-\frac{1}{p^{2}R}\right)\frac{\Delta_{E}}{\alpha_{T}\left(1-\nu \right)}\nabla^{2}w-\frac{\Delta_{E}}{\alpha_{T}}\left(1-\mu_{0}\nabla^{2}\right)\nabla^{2}\Phi ,$$

where $c_{\;1}=\frac{-1+\sqrt{1-4R^{2}p^{2}}}{2R}$, and $c_{\;2}=\frac{-1-\sqrt{1-4R^{2}p^{2}}}{2R}$. Parameters $b_{1}$ and $b_{2}$ are undetermined coefficients.

Considering that heat conduction on the inner and outer surfaces of the cylindrical shell is extremely weak, i.e., ${\left.\frac{\partial \Theta }{\partial z}\right|}_{\pm \frac{h}{2}}=0$ [24,25,47,48,49]. The specific forms of $b_{1}$ and $b_{1}$ can be determined using Equation (25) as follows:

(27a) $$b_{1}=\frac{\Delta_{E}}{\alpha_{T}\left(1-\nu \right)}\left(\frac{\sinh\left(\frac{\lambda_{2}h}{2}\right)}{\lambda_{1}\sinh\left(\left(\lambda_{1}-\lambda_{2}\right)\frac{h}{2}\right)}\right),$$

(27b) $$b_{2}=-\frac{\Delta_{E}}{\alpha_{T}\left(1-\nu \right)}\left(\frac{\;\sinh\left(\frac{\lambda_{1}h}{2}\right)}{\lambda_{2}\sinh\left(\left(\lambda_{1}-\lambda_{2}\right)\frac{h}{2}\right)}\right).$$

Substituting Equations (26a,b) into Equation (24), the temperature field distribution of the cylindrical shell can be obtained:

(28) $$\Theta =\frac{\Delta_{E}}{\alpha_{T}\left(1-\nu \right)}f\;\;\nabla^{2}w-\frac{\Delta_{E}}{Eh\alpha_{T}}\left(1-\mu_{0}\nabla^{2}\right)\nabla^{2}\Phi ,$$

where

(29) $$f=\frac{\sinh\left(\frac{\lambda_{2}h}{2}\right)}{\lambda_{1}\sinh\left(\left(\lambda_{1}-\lambda_{2}\right)\frac{h}{2}\right)}e^{\lambda_{1}z}-\frac{\sinh\left(\frac{\lambda_{1}h}{2}\right)}{\lambda_{2}\sinh\left(\left(\lambda_{1}-\lambda_{2}\right)\frac{h}{2}\right)}e^{\lambda_{2}z}+z-\frac{1}{Rp^{2}}.$$

### 2.3. Analytical Solutions for the Motion Equation and TED

The deflection $w_{\left(x,\theta ,t\right)}$ under harmonic vibrations of a cylindrical shell can be written as [24,25]:

(30) $$w_{\left(x,\theta ,t\right)}=w_{\left(x,\theta \right)}e^{j\omega t},$$

For simplicity, the following calculations will be set, i.e., $w=w_{\left(x,\theta \right)}$.

Substituting Equations (10a–c) into Equation (12), one can obtain:

(31) $$A_{0}\nabla^{4}w+\frac{1}{1-\nu }\nabla^{2}M_{T}+\left(1-\mu_{0}\nabla^{2}\right)\nabla_{k}^{2}\Phi -\omega^{2}\rho h\frac{\partial^{2}w}{\partial t^{2}}=0,$$

where $\nabla^{4}=\frac{\partial^{4}}{\partial x^{4}}+\frac{2}{R^{2}}\frac{\partial^{4}}{\partial \theta^{2}\partial x^{2}}+\frac{1}{R^{4}}\frac{\partial^{4}}{\partial \theta^{4}}$, $\nabla^{2}=\frac{\partial^{2}}{\partial x^{2}}+\frac{1}{R^{2}}\frac{\partial^{2}}{\partial \theta^{2}}$, and $\nabla_{k}^{2}=\frac{1}{R}\frac{\partial^{2}}{\partial x^{2}}$.

Substituting Equations (8a–c) into Equation (13), one can obtain:

(32) $$\left(1-\mu_{0}\nabla^{2}\right)\nabla^{4}\Phi +\nabla^{2}N_{T}-Eh\nabla_{k}^{2}w=0.$$

Substituting Equation (30) into Equations (31) and (32), one can obtain:

(33a) $$\left(A_{0}+\Delta_{E}F_{\left(\omega \right)}\right)\nabla^{4}w+\left(1-\mu_{0}\nabla^{2}\right)\nabla_{k}^{2}\Phi -\left(1-\mu_{0}\nabla^{2}\right)\omega^{2}\rho h\frac{\partial^{2}w}{\partial t^{2}}=0,$$

(33b) $$\frac{\Delta_{E}G_{\left(\omega \right)}}{1+\Delta_{E}}\nabla^{4}w+\left(1-\mu_{0}\nabla^{2}\right)\nabla^{4}\Phi -\frac{Eh}{1+\Delta_{E}}\nabla_{k}^{2}w=0,$$

where

(34a) $$F_{\left(\omega \right)}=\frac{Eh^{3}}{12{\left(1-\nu \right)}^{2}}\left(1+\frac{12}{h^{3}}\left(\begin{array}{l} \frac{1}{\lambda_{1}}\frac{\sinh\left(\frac{\lambda_{2}h}{2}\right)}{\sinh\left(\left(\lambda_{1}-\lambda_{2}\right)\frac{h}{2}\right)}g_{\left(\lambda_{1}\right)}\\ -\frac{1}{\lambda_{2}}\frac{\sinh\left(\frac{\lambda_{1}h}{2}\right)}{\sinh\left(\left(\lambda_{1}-\lambda_{2}\right)\frac{h}{2}\right)}g_{\left(\lambda_{2}\right)} \end{array}\right)\right),$$

(34b) $$G_{\left(\omega \right)}=\frac{E}{1-\nu }\left(\frac{2\sinh\left(\frac{\lambda_{2}h}{2}\right)\sinh\left(\frac{\lambda_{1}h}{2}\right)}{\sinh\left(\left(\lambda_{1}-\lambda_{2}\right)\frac{h}{2}\right)}\left(\frac{1}{\lambda_{1}^{2}}-\frac{1}{\lambda_{2}^{2}}\right)-\frac{h}{p^{2}R}\right),$$

(34c) $$g_{\left(\lambda \right)}=\frac{h}{\lambda }\cosh\left(\frac{\lambda h}{2}\right)-\frac{2}{\lambda^{2}}\sinh\left(\frac{\lambda h}{2}\right)\;.$$

By combining Equations (32a,b), the following can be obtained:

(35) $$\left(A_{0}+\Delta_{E}F_{\left(\omega \right)}\right)\nabla^{8}w-\frac{\Delta_{E}G_{\left(\omega \right)}\nabla_{k}^{2}\nabla^{4}w}{1+\Delta_{E}}+\frac{Eh\nabla_{k}^{4}w}{1+\Delta_{E}}=\left(1-\mu_{0}\nabla^{2}\right)\omega^{2}\rho h\nabla^{4}w.$$

where $F_{\left(\omega \right)}$ and $G_{\left(\omega \right)}$ are functions of frequency ω. The parameters $\nabla^{8}$ and $\nabla^{4}$ in Equation (35) are given as follows:

(36a) $$\nabla^{8}=\frac{d^{8}}{dx^{8}}-\frac{4}{R^{2}}\frac{d^{6}}{dx^{6}}+\frac{6}{R^{4}}\frac{d^{4}}{dx^{4}}-\frac{4}{R^{6}}\frac{d^{2}}{dx^{2}}+\frac{1}{R^{8}},$$

(36b) $$\nabla^{4}=\frac{d^{4}}{dx^{4}}-\frac{2}{R^{2}}\frac{d^{2}}{dx^{2}}+\frac{1}{R^{4}}.$$

To obtain the explicit expression for the frequency of the cylindrical shell, thermoelastic coupling behavior is neglected by setting, i.e., *α_T_* = 0 [24,25,48,49]. In this case, Equation (35) can be reduced to:

(37) $$A_{0}\nabla^{8}w+Eh\nabla_{k}^{4}w=\left(1-\mu_{0}\nabla^{2}\right)\omega_{0}^{2}\rho h\nabla^{4}w,$$

where $\omega_{0}$ represents the isothermal frequency of the cylindrical shell.

For a cylindrical shell that is closed in the θ direction, the solution to Equation (35) satisfies the following relationship [24,49]:

(38) $$w=w_{m(x)}\cos n\left(\theta -\varphi \right)\;,$$

where φ represents any angle.

Substituting Equations (38) into (37) and using the Galerkin method, the expression for the isothermal frequency $\omega_{0}$ can be obtained:

(39) $$\omega_{0}=\sqrt{\frac{A_{0}\int_{0}^{L}w_{m}\nabla^{8}w_{m}dx+Eh\int_{0}^{L}w_{m}\nabla_{k}^{4}w_{m}dx}{\rho h\left(1-\mu_{0}\nabla^{2}\right)\int_{0}^{L}w_{m}\nabla^{4}w_{m}dx}}.$$

When the size effect is neglected, i.e., $\mu_{0}=0$, $\tau_{T}=0$, $\tau_{q}=0$, and $l_{Q}=0$ the isothermal frequency of the cylindrical shell, namely Equation (36) in the manuscript, can be degenerated into:

(40) $$\omega_{0}^{2}=\frac{1}{\rho h}\left(D{\left({\left(\frac{n}{R}\right)}^{2}+\lambda_{m}^{2}\right)}^{2}+\frac{Eh\lambda_{m}^{4}}{R^{2}{\left({\left(\frac{n}{R}\right)}^{2}+\lambda_{m}^{2}\right)}^{2}}\right).$$

where $D=\frac{Eh^{3}}{12\left(1-\nu^{2}\right)}$. Equation (40) is consistent with Equation (41) in reference [28].

Due to the weak thermoelastic coupling effect, $F_{\left(\omega_{0}\right)}$ and $G_{\left(\omega_{0}\right)}$ can be used to approximately replace $F_{\left(\omega \right)}$ and $G_{\left(\omega \right)}$ in Equation (35). At this point, Equation (35) can be written as:

(41) $$\left(A_{0}+\Delta_{E}F_{\left(\omega_{0}\right)}\right)\nabla^{8}w-\frac{\Delta_{E}G_{\left(\omega_{0}\right)}\nabla_{k}^{2}\nabla^{4}w}{1+\Delta_{E}}+\frac{Eh\nabla_{k}^{4}w}{1+\Delta_{E}}=\left(1-\mu_{0}\nabla^{2}\right)\omega^{2}\rho h\nabla^{4}w.$$

Based on the Galerkin method, the expression for the frequency ω of the cylindrical shell can be obtained from Equation (40) as:

(42) $$\omega =\psi_{1}+\psi_{2}j,$$

where $\psi_{1}=\sqrt{\frac{\omega_{r}+\sqrt{\omega_{r}^{2}+\omega_{i}^{2}}}{2}}$, and $\psi_{2}=\sqrt{\frac{-\omega_{r}+\sqrt{\omega_{r}^{2}+\omega_{i}^{2}}}{2}}$, in which $\omega_{r}$ and $\omega_{i}$ represent the real part and imaginary part of the square of the cylindrical shell frequency, respectively. The expressions for both the real part and the imaginary part mentioned above are as follows:

(43a) $$\omega_{r}=\omega_{r1}-\omega_{r2}+\omega_{r3},$$

(43b) $$\omega_{i}=\frac{\Delta_{E}F_{i}}{\rho h}\left(\frac{\int_{0}^{L}w_{m}\nabla^{8}w_{m}dx}{\int_{0}^{L}w_{m}\nabla^{4}w_{m}dx}\right)-\frac{\Delta_{E}G_{i}}{\rho h\left(1+\Delta_{E}\right)}\left(\frac{\int_{0}^{L}w_{m}\nabla_{k}^{2}\nabla^{4}w_{m}dx}{\int_{0}^{L}w_{m}\nabla^{4}w_{m}dx}\right),$$

where $\omega_{r1}=\frac{\left(A_{2}+\Delta_{E}F_{r}\right)}{\rho h}\left(\frac{\int_{0}^{L}w_{m}\nabla^{8}w_{m}dx}{\int_{0}^{L}w_{m}\nabla^{4}w_{m}dx}\right)$, $\omega_{r2}=\frac{\Delta_{E}G_{r}}{\rho h\left(1+\varepsilon \right)}\left(\frac{\int_{0}^{L}w_{m}\nabla_{k}^{2}\nabla^{4}w_{m}dx}{\int_{0}^{L}w_{m}\nabla^{4}w_{m}dx}\right)$, and $\omega_{r3}=\frac{A_{1}}{\rho h\left(1+\varepsilon \right)}\left(\frac{\int_{0}^{L}w_{m}\nabla_{k}^{4}w_{m}dx}{\int_{0}^{L}w_{m}\nabla^{4}w_{m}dx}\right)$. Parameter $F_{r}$ is the real part of the function $F_{\left(\omega_{0}\right)}$, $F_{i}$ is the imaginary part of the function $F_{\left(\omega_{0}\right)}$, $G_{r}$ is the real part of the function $G_{\left(\omega_{0}\right)}$, and $G_{i}$ is the imaginary part of the function $G_{\left(\omega_{0}\right)}$.

Substituting Equations (42) and (43a,b) into the formula for TED:

(44) Q−1=2ImωReω ,

One can obtain:

(45) $$Q^{-1}=2\left|\frac{\omega_{i}}{\omega_{r}+\sqrt{\omega_{r}^{2}+\omega_{i}^{2}}}\right|\;.$$

Based on the weak thermoelastic coupling assumption commonly used in TED analysis. Under this assumption, the energy loss caused by thermoelasticity is much smaller than the stored mechanical energy, and the influence of the thermal field on the real part of the resonant frequency is considered a small disturbance. Considering the weaker effect of thermoelastic coupling, i.e., $\omega_{i}\ll \omega_{r}$ [48,49], Equation (45) can be simplified to:

(46) $$Q^{-1}=\left|\frac{\omega_{i}}{\omega_{r}}\right|\;.$$

Substituting Equations (43a,b) into Equation (46) and taking into account $\Delta_{E}\ll 1$, Equation (46) can be specifically expressed as:

(47) $$Q^{-1}=\left|\frac{\Delta_{E}F_{i}\int_{0}^{L}w_{m}\nabla^{8}w_{m}dx-\frac{\Delta_{E}G_{i}}{1+\varepsilon }\int_{0}^{L}w_{m}\nabla_{k}^{2}\nabla^{4}w_{m}dx}{A_{2}\int_{0}^{L}w_{m}\nabla^{8}w_{m}dx+\frac{A_{1}}{1+\varepsilon }\int_{0}^{L}w_{m}\nabla_{k}^{4}w_{m}dx}\right|\;.$$

It should be noted that when the mechanical nonlocal parameter and the thermal nonlocal parameter are set to zero, the present model can degenerate into the classical TED model.

## 3. TED Expression of Micro/Nano Cylindrical Shells Under Different Boundary Conditions

According to Yu’s hypothesis [51,52], the transverse vibration of a cylindrical shell can be approximately described by the transverse vibration equation of a beam. The free vibration equation of an Euler–Bernoulli beam in the context of NET is expressed as follows [41,48]:

(48) $$EI\frac{\partial^{4}w}{\partial x^{4}}-\mu_{0}\rho A\frac{\partial^{4}w}{\partial x^{2}\partial t^{2}}+\rho A\frac{\partial^{2}w}{\partial t^{2}}=0,$$

where I and *A* represent the area moment of inertia of cross-sections and cross-sectional area of the beam. It should be noted that for thick shells, high-order modes, or complex boundary conditions, more refined shell theory or numerical eigenfunctions may be required.

Under harmonic vibration, the deflection of the beam must satisfy the relationship $w_{\left(x,t\right)}=w_{m\left(x\right)}e^{j\omega t}$. Substituting this expression into Equation (48), one can obtain:

(49) $$\frac{\partial^{4}w_{m}}{\partial x^{4}}+\mu_{0}\lambda_{m}^{4}\frac{\partial^{4}w_{m}}{\partial x^{4}}-\lambda_{m}^{4}w_{m}=0,$$

where $\lambda_{m}^{4}=\frac{\rho A}{EI}\omega^{2}$.

The solution of Equation (49) has the following form [41,48]:

(50) $$w_{m}=N_{1}\cos\left(r_{1}x\right)+N_{2}\sin\left(r_{1}x\right)+N_{3}\cosh\left(r_{2}x\right)+N_{4}\sinh\left(r_{2}x\right),$$

where $N_{1}$, $N_{2}$, $N_{3}$, and $N_{4}$ are the integration constants, and $w_{m}$ is the mode shape function.

Substituting Equation (44) into Equation (49), one can obtain:

(51) $$\lambda_{m}^{4}=\frac{r_{1}^{4}}{1+\mu_{0}r_{1}^{2}}=\frac{r_{2}^{4}}{1-\mu_{0}r_{2}^{2}}.$$

The classical boundary conditions widely used in practical engineering were investigated as fully clamped (C-C), fully simply supported (S-S), and clamped-free (C-F) conditions:

(52) $$S\;S\;:w_{m\left(0\right)}=\frac{d^{2}w_{m}}{dx^{2}}\left(0\right)=w_{m\left(L\right)}=\frac{d^{2}w_{m}}{dx^{2}}\left(L\right)=0\;,$$

(53) $$C\;C\;:w_{m\left(0\right)}=\frac{dw_{m}}{dx}\left(0\right)=w_{m\left(L\right)}=\frac{dw_{m}}{dx}\left(L\right)=0\;,$$

(54) $$\begin{array}{l} C\;F\;:w_{m\left(0\right)}=\frac{dw_{m}}{dx}\left(0\right)=EI\;\frac{d^{2}w_{m}}{dx^{2}}\left(L\right)+\mu_{0}\rho A\omega_{m}^{2}w_{m\left(L\right)}\\ \;\;\;\;\;\;=EI\;\frac{d^{3}w_{m}}{dx^{3}}\left(L\right)+\mu_{0}\rho A\omega_{m}^{2}\frac{dw_{m}}{dx}\left(L\right)=0. \end{array}$$

By substituting Equation (50) into Equations (52)–(54) and setting the determinant of the coefficient matrix of the algebraic equations for $N_{1}$, $N_{2}$, $N_{3}$, and $N_{4}$ to zero, the following relationship is obtained:

(55) $$S\;S\;:\sin\left(r_{1}L\right)=0,$$

(56) $$C\;C\;:2\cos\left(r_{1}L\right)\cosh\left(r_{2}L\right)+\left(\frac{r_{1}}{r_{2}}-\frac{r_{2}}{r_{1}}\right)\sin\left(r_{1}L\right)\sinh\left(r_{2}L\right)-2=0,$$

(57) $$\begin{array}{l} C\;F\;:2\cos\left(r_{1}L\right)\cosh\left(r_{2}L\right)+\left(\frac{r_{1}}{r_{2}}-\frac{r_{2}}{r_{1}}\right)\sin\left(r_{1}L\right)\sinh\left(r_{2}L\right)\\ \;\;\;+\left(\frac{r_{1}^{2}}{r_{2}^{2}}+\frac{r_{2}^{2}}{r_{1}^{2}}\right)=0. \end{array}$$

It should be noted that *r*_1_ and *r*_2_ are interrelated with Equation (51). By solving Equations (55)–(57), the shape function $w_{m}$ of the nonlocal beam with the specified boundary conditions can be obtained. The mechanical boundary conditions determine the vibration mode shapes of the structure, while the thermal boundary conditions govern the temperature distribution and heat conduction process. The coupling between the stress field and the temperature field is achieved through the thermoelastic source terms related to strain or volumetric deformation.

Substituting the shape function $w_{m}$ under the boundary conditions into Equation (46) yields the TED expression of the cylindrical shell with size effects for the corresponding boundary conditions:

(58) $$Q^{-1}=\frac{\Delta_{E}F_{i}{\left({\left(\frac{n}{R}\right)}^{2}+\lambda_{m}^{2}\right)}^{4}-\frac{\Delta_{E}G_{i}}{R\left(1+\varepsilon \right)}{\left({\left(\frac{n}{R}\right)}^{2}+\lambda_{m}^{2}\right)}^{2}}{A_{2}{\left({\left(\frac{n}{R}\right)}^{2}+\lambda_{m}^{2}\right)}^{4}+\frac{A_{1}\lambda_{m}^{4}}{R^{2}\left(1+\varepsilon \right)}}\;.$$

## 4. Discussion of Numerical Results

This section includes four parts: the first part is the validation of numerical results; the second part investigates the influence of size effects on the TED of cylindrical shells at the micro/nanoscale; the third part analyzes the impact of size effects on the FS of cylindrical shells at the micro/nanoscale; the fourth part explores the influence of size effects on the FA of cylindrical shells at the micro/nanoscale. The numerical analysis uses a micro/nano cylindrical shell resonator made of silicon as an example. The material parameters for silicon are shown in Table 1. Unless otherwise specified, the values of the parameters are as follows: h=25 nm, R/h=10, L/R=5, $\mu_{0}=2\;{\mathrm{nm}}^{2}$, $T_{0}=300\;K$, $\tau_{q}=33.3\;\mathrm{ps}$, $\tau_{T}=1.66\;\mathrm{ps}$, $l_{Q}=30\;\mathrm{nm}$, n=2 and m=2 [49]. It should be noted that directly identifying mechanical and thermal nonlocal effects through experiments in micro nano cylindrical shells is still challenging. Existing experimental studies typically measure the overall resonant frequency, frequency shift, or quality factor of nano resonators, but it is still difficult to independently separate the contributions of mechanical and thermal nonlocality. Therefore, in many theoretical studies, non-local parameters are considered as feature length scale parameters and parameterized within physically meaningful ranges. Mechanical nonlocal parameters reflect the long-range interactions and intrinsic length scales of materials at the micro- and nanoscale, while thermal nonlocal parameters characterize the spatial nonlocal thermal conduction when the structural characteristic size is equal to the phonon mean free path. Therefore, the selected nonlocal parameter values are not arbitrarily determined, but are used to characterize possible nanoscale effects.

### 4.1. Validation of Numerical Results

**Figure 2:**
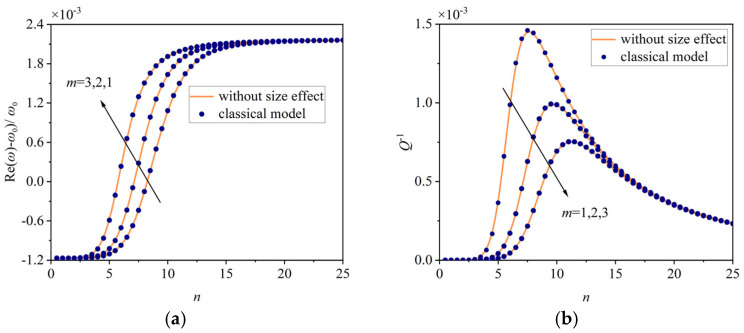
Numerical verification ($h/R=3.33\times 10^{-3}$, R/L=0.25, and $R=76.2\times 10^{-3}\;m$): (**a**) FS of a cylindrical steel shell with C-C boundary conditions, and (**b**) TED in a cylindrical steel shell with C-C boundary conditions.

To validate the proposed theoretical model, Figure 2a,b compares its calculated results with the numerical data from Lu et al. [24], neglecting size effects. Figure 2a,b shows how the TED and FS of a steel cylindrical shell change with the circumferential mode number n under C-C boundary conditions. The material parameters of steel are detailed in Table 2. As shown in Figure 2a, the TED of the cylindrical shell changes regularly with the mode number *n*. A significant damping peak is also observed. Figure 2b reflects the evolution of the FS. When *n* < 5, the mode number weakly affects the FS. For 5 ≤ *n* ≤ 15, the FS fluctuates drastically with n. When *n* > 15, the FS approaches a stable constant, making the influence of n negligible. The results indicate that when size effects are neglected, the predictions of the present model are identical to the numerical solutions of the classical theoretical model. Due to the limited direct experimental verification of NDPL models for cylindrical nano shell resonators, this study mainly focuses on theoretical modeling and parameter analysis.

### 4.2. Influence of Size Effect on the TED of Cylindrical Shells

**Figure 3:**
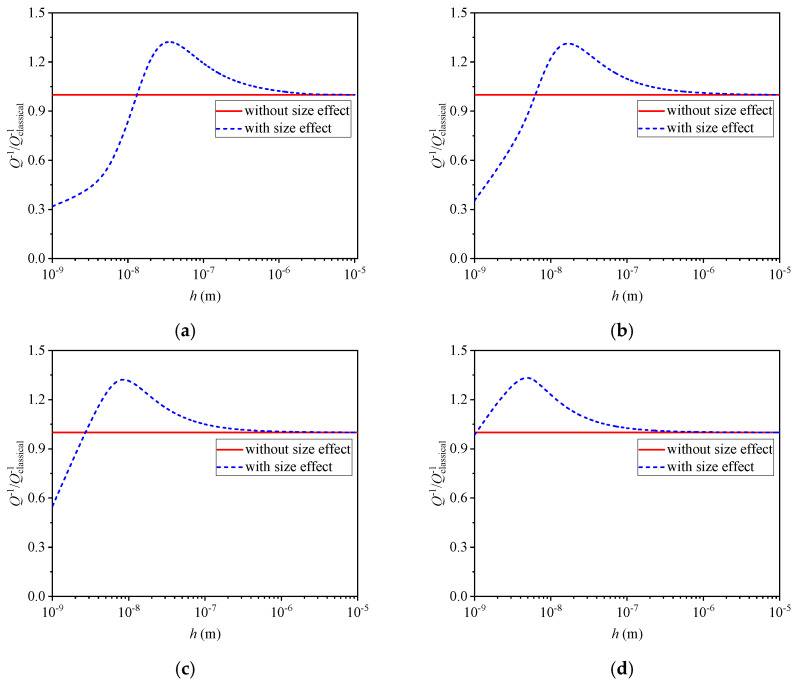
Influence of the size effect on TED ($\mu_{0}\neq 0,\;\tau_{T}\neq 0,\;\tau_{q}\neq 0\;\;\mathrm{and}\;l_{Q}\neq 0$): (**a**) R/h=10, (**b**) R/h=20, (**c**) R/h=40, and (**d**) R/h=80.

Figure 3a–d reveals the influence of size effects on the TED of cylindrical shells. It should be emphasized that the size effects in this study account for both mechanical nonlocal effects and non-Fourier heat conduction behavior. Figure 3a–d shows the results for radius-to-thickness ratios (*R*/*h*) of 10, 20, 40, and 80. Compared with classical theoretical predictions, the TED of cylindrical shells considering size effects first increases and then decreases as the shell thickness increases. Further observation reveals that this evolution trend becomes increasingly significant as the radius-to-thickness ratio decreases. Specifically, when the shell thickness decreases to the nanoscale, the impact of size effects on TED gradually emerges. Moreover, as the radius-to-thickness ratio of the cylindrical shell decreases, this size dependence becomes increasingly pronounced. In summary, the TED of cylindrical shells at the nanoscale exhibits significant size-dependent characteristics. Traditional classical thermoelastic models fail to effectively predict this physical phenomenon because they ignore microscopic size parameters.

**Figure 4:**
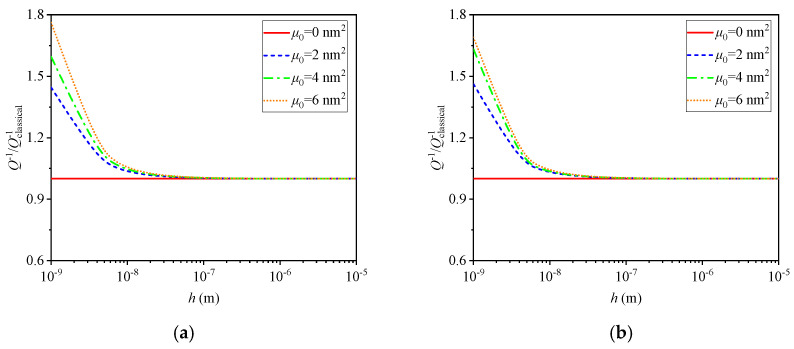

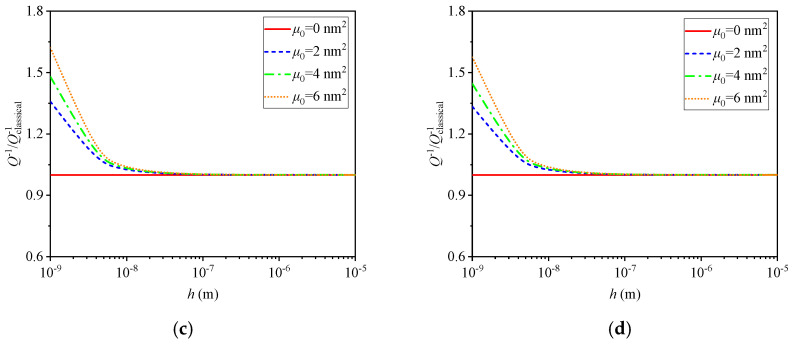
Influence of the nonlocal parameter $\mu_{0}$ on TED: (**a**) R/h=10, (**b**) R/h=20, (**c**) R/h=40, and (**d**) R/h=80.

Figure 4a–d describes the influence of the mechanical nonlocal effect on the TED of cylindrical shells. During the numerical simulation, the influence of the mechanical nonlocal effect on TED is analyzed independently by adjusting the magnitude of the mechanical nonlocal parameter $\mu_{0}$. In the calculations $\tau_{T}=0$, $\tau_{q}=0$, and $l_{Q}=0$. Figure 4a–d shows that the mechanical nonlocal effect significantly increases the TED of the cylindrical shell at the nanoscale. Meanwhile, the impact of mechanical nonlocality on TED becomes more pronounced as the mechanical nonlocal parameter $\mu_{0}$ increases. Additionally, by comparing Figure 4a–d, it can be found that the influence of the mechanical nonlocal effect on TED gradually weakens as the radius-to-thickness ratio increases.

**Figure 5:**
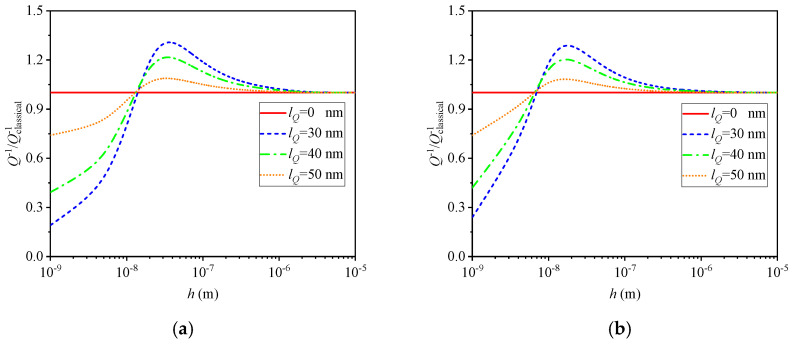

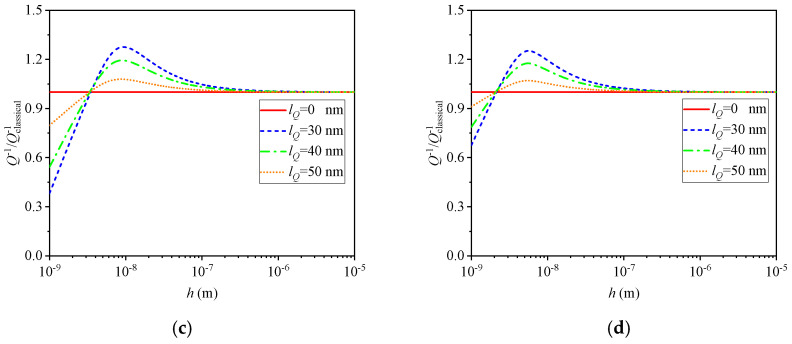
Influence of the thermal nonlocal parameter $l_{Q}$ on TED: (**a**) R/h=10, (**b**) R/h=20, (**c**) R/h=40, and (**d**) R/h=80.

Figure 5a–d describes the influence of non-Fourier heat conduction behavior on the TED of cylindrical shells. During the numerical simulation, the influence of the mechanical nonlocal effect on TED is ignored by setting $\mu_{0}=0$. The results show that as the shell thickness decreases, the TED first increases and then decreases compared to classical models when considering the non-Fourier effect. Moreover, it exhibits obvious differences at the nanoscale. Additionally, as the thermal nonlocal parameter $l_{Q}$ increases, the numerical results for TED gradually decrease. The thermal nonlocal $l_{Q}$ parameter represents the mean free path of heat carriers. This refers to the average distance heat carriers travel when transferring energy within a solid. When the thermal nonlocal parameter increases, the collision probability among heat carriers decreases. This reduces the energy loss caused by these collisions, thereby decreasing the TED of the cylindrical shells. Finally, as the radius-to-thickness ratio of the cylindrical shell increases, the peak of TED gradually shifts to the left. This phenomenon indicates the impact of structural geometric properties on TED behavior. It further verifies the complexity of non-Fourier heat conduction at the nanoscale.

To summarize, mechanical nonlocality reflects the long-range interaction between material points at the micro/nanoscale. In the framework of nonlocal elasticity, the stress at a point depends not only on the strain at that point but also on the strain field in its surrounding region. This effect generally reduces effective structural stiffness, enhances deformation and strain redistribution, and consequently strengthens the thermoelastic coupling. As a result, the thermoelastic energy dissipation tends to increase with the mechanical nonlocal parameter. By contrast, thermal nonlocality modifies the heat conduction process when the structural characteristic length becomes comparable to the phonon mean free path. The nonlocal thermal effect changes the relation between the temperature gradient and heat flux and may weaken the effective local heat diffusion associated with thermoelastic damping. Therefore, the irreversible heat flow caused by strain-induced temperature gradients can be reduced, leading to a decrease in TED. The observed TED behavior is therefore governed by the competition between these two size-dependent mechanisms. Mechanical nonlocality tends to enhance TED through effective stiffness reduction and stronger strain-temperature coupling, whereas thermal nonlocality tends to suppress TED through modified heat conduction. This competing mechanism is important for resonator design. For high-Q resonators, controlling the shell geometry, material properties, and thermal transport characteristics can help reduce TED and improve the quality factor.

### 4.3. The Influence of Size Effect on FS of Cylindrical Shells

**Figure 6:**
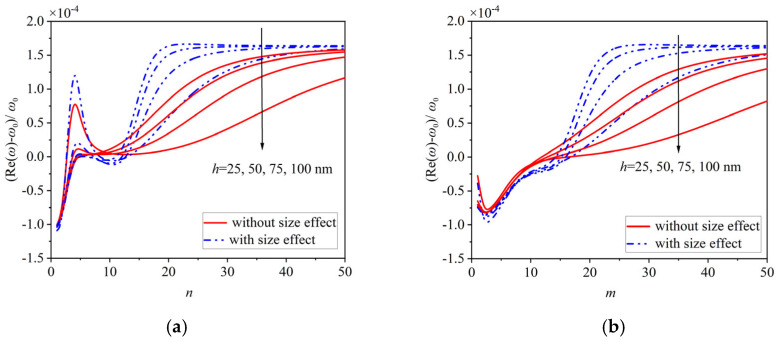
The influence of size effects on FS Reω−ω0/ω0, $\mu_{0}\neq 0$, $\tau_{T}\neq 0$, $\tau_{q}\neq 0$, and $l_{Q}\neq 0$: (**a**) circumferential vibration modal orders *n*, and (**b**) transverse vibration modal orders *m*.

Figure 6a,b describes the influence of size effects on the FS of the cylindrical shell at a radius-to-thickness ratio of *R*/*h* = 20. It can be observed from Figure 6a that under the circumferential vibration mode, the size effect increases the FS amplitude of the cylindrical shell when the mode number *n* is between 1 and 8. When the circumferential vibration mode number *n* is between 8 and 12, the size effect decreases the FS amplitude. When the circumferential vibration mode number *n* exceeds 12, the size effect increases the FS amplitude again. However, in the transverse vibration mode *m*, Figure 6b shows that as the transverse mode number *m* increases, the size effect initially decreases the FS amplitude. Subsequently, when *m* exceeds 18, the size effect increases the amplitude of the FS of the cylindrical shell. It is evident that the influence of size effects on the FS of the cylindrical shell varies under different vibration modes.

**Figure 7:**
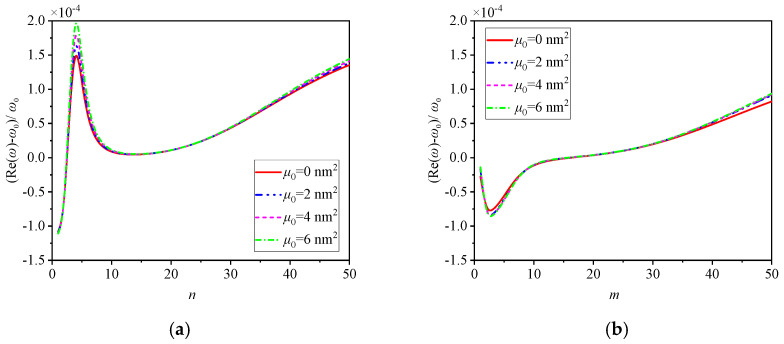
Influence of the nonlocal parameter $\mu_{0}$ on FS Reω−ω0/ω0, $\tau_{T}=0$, $\tau_{q}=0$, and $l_{Q}=0$: (**a**) circumferential vibration modal orders *n*, and (**b**) transverse vibration modal orders *m*.

Figure 7a,b shows the influence of the mechanical nonlocal parameter $\mu_{0}$ on the FS of the cylindrical shell when the thickness is *h* = 25, and the radius-to-thickness ratio is *R*/*h* = 20. At this time, it should be noted that the effect of non-Fourier heat conduction on the FS is neglected. In the calculations $\tau_{T}=0$, $\tau_{q}=0$ and $l_{Q}=0$. It can be observed from Figure 7a that when the circumferential vibration mode number *n* is in the range of 1 to 10, the amplitude of the FS gradually increases as the mechanical nonlocal parameter increases. However, when *n* increases to the range of 10 to 35, the influence of the mechanical nonlocal parameter on the FS is relatively small. When *n* exceeds 35, the influence of the mechanical nonlocal parameter on the FS gradually strengthens again. It can be seen in Figure 7b that under different transverse mode numbers *m*, the influence of the mechanical nonlocal effect on the FS is very similar to the trend under the circumferential vibration mode. These results indicate that the mechanical nonlocal parameter significantly affects the FS characteristics of the cylindrical shell.

**Figure 8:**
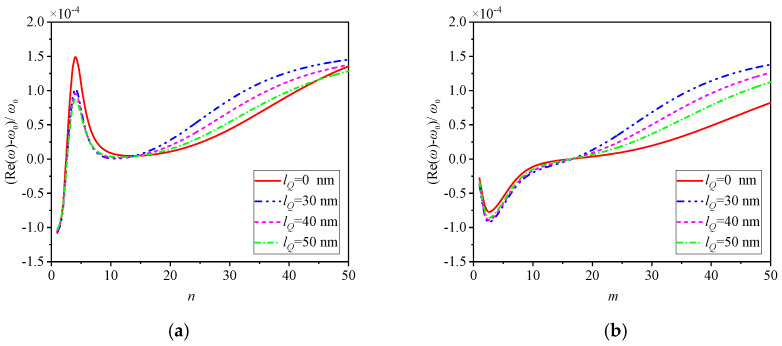
Influence of the thermal nonlocal parameter $l_{Q}$ on FS: Reω−ω0/ω0, $\mu_{0}=0$: (**a**) circumferential vibration modal orders *n*, and (**b**) transverse vibration modal orders *m*.

Figure 8a,b reveals the influence of the thermal nonlocal parameter $\mu_{0}$ on the FS of the cylindrical shell when the thickness is *h* = 25, and the radius-to-thickness ratio is *R*/*h* = 20. Notably, the mechanical nonlocal effect is ignored here, meaning the corresponding parameter is set to zero, i.e., $\mu_{0}=0$. Figure 8a shows that when the circumferential vibration mode number *n* is between 1 and 15, the FS amplitude gradually decreases as the thermal nonlocal parameter increases. However, when *n* exceeds 15, the thermal nonlocal effect will alter the FS amplitude of the cylindrical shell. Furthermore, as the thermal nonlocal parameter increases, the FS amplitude will decrease. Figure 8b shows that under transverse vibration modes, the influence of the thermal nonlocal effect on the FS is similar to that under circumferential vibration modes. These results demonstrate that the thermal nonlocal parameter significantly affects the FS characteristics of the cylindrical shell. Additionally, this specific effect is highly dependent on the vibration mode number.

In summary, the size effect causes a significant change in the FS amplitude of the cylindrical shell. Furthermore, it exhibits similar effects across different vibration modes. This emphasizes the need to fully consider the size effect when designing and optimizing nano-resonators.

### 4.4. The Influence of Size Effect on FA of Cylindrical Shells

**Figure 9:**
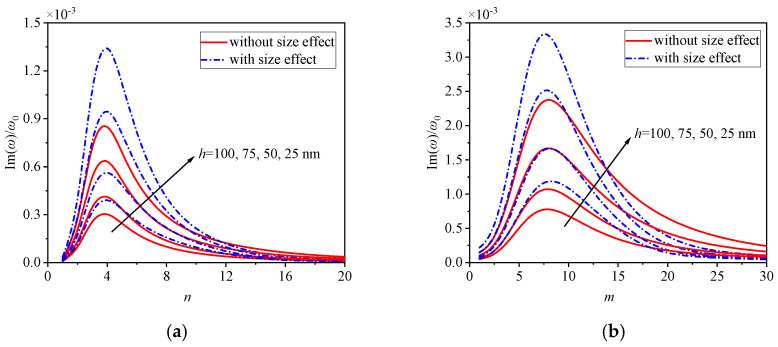
Influence of the size effects on FA Imω/ω0, $\mu_{0}\neq 0$, $\tau_{T}\neq 0$, $\tau_{q}\neq 0$, and $l_{Q}\neq 0$: (**a**) circumferential vibration modal orders *n*, and (**b**) transverse vibration modal orders *m*.

Figure 9a,b presents the relationship between the FA of the cylindrical shell and its circumferential mode number *n* and transverse mode number *m*. It can be seen from Figure 9a,b that the FA of the cylindrical shell exhibits a clear peak. For example, in the circumferential vibration mode, the FA reaches its maximum when *n* ≈ 4. In contrast, for the transverse vibration mode, the FA also reaches its maximum when *m* ≈ 7. In addition, Figure 9a,b indicates that size effects significantly increase the amplitude of FA in the cylindrical shell. This finding is of great significance for improving the accuracy of nano-resonators. Finally, by comparing Figure 9a,b, it is clear that the effect of the transverse vibration mode on FA is significantly greater than that of the circumferential vibration mode. This indicates that greater attention should be paid to FA under transverse vibration modes when designing and optimizing the vibration characteristics of cylindrical-shell resonators.

**Figure 10:**
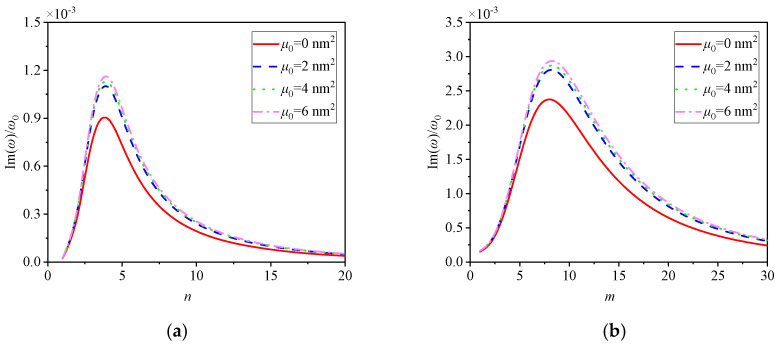
Influence of the nonlocal parameter $\mu_{0}$ on FA Imω/ω0, $\tau_{T}=0$, $\tau_{q}=0$, and $l_{Q}=0$: (**a**) circumferential vibration modal orders *n*, and (**b**) transverse vibration modal orders *m*.

Figure 10a,b shows the influence of the mechanical nonlocal parameter on the FA of the cylindrical shell when the shell thickness is *h* = 25, and the radius-to-thickness ratio is *R*/*h* = 20. It should be emphasized that non-Fourier heat conduction is neglected here, i.e., $\tau_{T}=0$, $\tau_{q}=0$ and $l_{Q}=0$ is set. It can be seen from Figure 10a,b that the peak value of FA gradually increases as the mechanical nonlocal parameter increases. In addition, the effect of thermal nonlocal behavior on the FA of the cylindrical shell exhibits a similar trend under different vibration modes. These results show that the mechanical nonlocal effect influences the FA characteristics of the cylindrical shell. In practical applications, this is important for optimizing the vibration characteristics of cylindrical shells.

**Figure 11:**
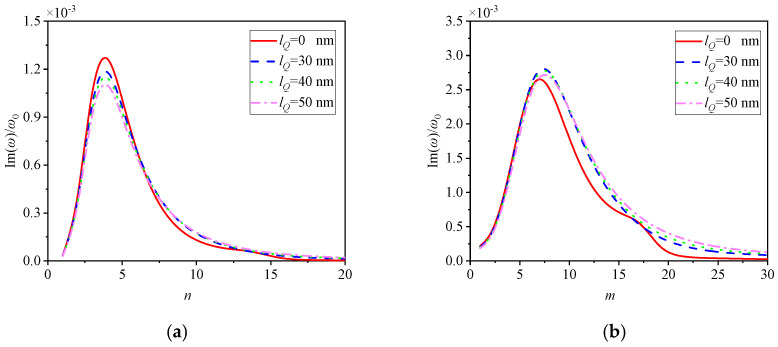
Influence of the thermal nonlocal parameter $l_{Q}$ on FA Imω/ω0, $\mu_{0}=0$: (**a**) circumferential vibration modal orders *n*, and (**b**) transverse vibration modal orders *m*.

Figure 11a,b shows the effect of the thermal nonlocal parameter on the FA of the cylindrical shell when the shell thickness is *h* = 25, and the radius-to-thickness ratio is *R*/*h* = 20. It should be emphasized that the mechanical nonlocal effect is neglected in this analysis, i.e., $\mu_{0}=0$. Figure 11a shows that under the circumferential vibration mode, the FA amplitude of the cylindrical shell gradually decreases as the thermal nonlocal parameter increases. In contrast, Figure 11b indicates that under the transverse vibration mode, the FA amplitude of the cylindrical shell increases as the thermal nonlocal parameter increases. These results indicate that the influence of the thermal nonlocal effect on the FA of the cylindrical shell differs significantly among vibration modes. This provides an important basis for the design and optimization of cylindrical shells.

In summary, the effect of size dependence on the FA of cylindrical shells differs significantly across vibration modes.

## 5. Conclusions Remarks

Based on the nonlocal elasticity theory and the NDPL model, this paper establishes a theoretical model for the TED of micro/nano cylindrical shells using the DMV approximation. The analytical solution for the TED of cylindrical shells under classical boundary conditions is obtained using the complex frequency method. The influencing factors of TED in nanoscale cylindrical shells are investigated. Furthermore, the effects of size dependence on FA and FS are analyzed. The numerical results indicate that:

(1) Compared to the numerical results predicted by the classical theory, the TED of the cylindrical shell exhibits a high degree of size dependence when size effects are considered. This size dependence is particularly pronounced at the nanoscale. Classical thermoelastic theory cannot effectively predict this phenomenon.

(2) At the nanoscale, the mechanical nonlocal effect significantly enhances the TED of the cylindrical shell. This is because the mechanical nonlocal effect leads to material softening, thereby reducing its bending stiffness. As the mechanical nonlocal parameter increases, its impact on the TED of the cylindrical shell becomes more pronounced. Furthermore, as the radius-to-thickness ratio increases, the influence of the mechanical nonlocal effect gradually weakens.

(3) An increase in the thermal nonlocal parameter reduces the TED amplitude of the cylindrical shell. This is related to the decreased collision probability among heat carriers.

(4) The effect of the mechanical nonlocal parameter on the FS of cylindrical shells shows significant differences at different mode orders. In the circumferential vibration mode and at lower mode orders, an increase in the mechanical nonlocal parameter gradually enlarges the amplitude of FS. In contrast, under the transverse vibration mode, an increase in the mechanical nonlocal parameter gradually decreases the amplitude of FS.

(5) The effect of the thermal nonlocal parameter on the FA of cylindrical shells shows significant differences across vibration modes. Specifically, under the circumferential vibration mode, the thermal nonlocal parameter reduces the FA of the cylindrical shell. In contrast, under the transverse vibration mode, the thermal nonlocal parameter increases the FA of the cylindrical shell.

In summary, the TED of nano cylindrical shells changes significantly with size variation. This phenomenon cannot be accurately predicted by classical TED models. These findings provide a theoretical basis for designing high-Q nano cylindrical shell resonators, particularly in gyroscopic and sensing applications.

## Figures and Tables

**Table 1:** Physical constants of silicon [49].

$T_{0}\left(K\right)$	$E\left(\mathrm{GPa}\right)$	***ν***	$\rho \;\left({\mathrm{kgm}}^{-3}\right)$	$\alpha_{T}\left(10^{-6}K^{-1}\right)$	$C_{E}\left(m^{-3}K^{-1}\right)$	$\kappa \left({\mathrm{Wm}}^{-1}K^{-1}\right)$
300	160.0	0.22	2300	2.6	695	150

**Table 2:** Physical constants of steel [24].

$T_{0}\left(K\right)$	$E\left(\mathrm{GPa}\right)$	ν	$\rho \;\left({\mathrm{kgm}}^{-3}\right)$	$\alpha_{T}\left(10^{-6}K^{-1}\right)$	$C_{E}\left(m^{-3}K^{-1}\right)$	$\kappa \left({\mathrm{Wm}}^{-1}K^{-1}\right)$
300	206	0.3	7850	12	484	52

## Data Availability

The original contributions presented in this study are included in the article. Further inquiries can be directed to the corresponding author.
